# Lipoprotein(a) and Vascular Redox State in Patients With Advanced Coronary Atherosclerosis

**DOI:** 10.1161/ATVBAHA.125.322924

**Published:** 2025-10-30

**Authors:** Murray D. Polkinghorne, Ileana Badi, Andrea Baragetti, Jagat Chauhan, Cheng Xie, Elizabeth Wahome, Ioannis Akoumianakis, Daniel Foran, Parijat Patel, Edmarcia de Araujo, Christos P. Kotanidis, George Krasopoulos, Rana Sayeed, Vivek Srivastava, Antonios Kourliouros, Nicholas Walcot, Priya Sastry, Tomasz Guzik, Keith M. Channon, Giuseppe D. Norata, Charalambos Antoniades

**Affiliations:** 1 Division of Cardiovascular Medicine, Radcliffe Department of Medicine (M.D.P., I.B., J.C., C.X., E.W., I.A., D.F., P.P., E.d.A., C.P.K., K.M.C., C.A.), University of Oxford, United Kingdom.; 2 Division of Cardiovascular Medicine, Acute Multidisciplinary Imaging and Interventional Centre (C.A.), University of Oxford, United Kingdom.; 3 Department of Metabolism, Digestion, and Reproduction, Imperial College London, United Kingdom (M.D.P.).; 4 Department of Excellence of Pharmacological and Biomolecular Sciences, University of Milan, Italy (A.B., G.D.N.).; 5 John Radcliffe Hospital, Oxford University Hospitals, United Kingdom (G.K, R.S., V.S., A.K., N.W., P.S.).; 6 Centre for Cardiovascular Sciences, The University of Edinburgh, United Kingdom (T.G.).

**Keywords:** atherosclerosis, coronary artery disease, inflammation, lipoprotein(a), risk factors

## Abstract

**BACKGROUND:**

Lp(a) (lipoprotein[a]) is associated with cardiovascular disease, but neither the causal nature nor the underlying mechanisms are fully documented. This study investigated whether Lp(a) triggers atherogenesis by dysregulating vascular redox–sensitive inflammatory state.

**METHODS:**

Plasma Lp(a) was measured in 1027 patients with advanced coronary artery disease undergoing cardiac surgery. These patients were genotyped, and a modified *LPA* genetic risk score (LPA GRS) determining Lp(a) levels was generated. RNA sequencing and vascular superoxide measurements were performed in internal mammary arteries, and the contribution of NOXs (NADPH oxidases) and uncoupled eNOS (endothelial nitric oxide synthase) was determined. The median follow-up was 5.07 years.

**RESULTS:**

Increased plasma Lp(a) (*P*=0.03) and LPA GRS (*P*=0.01) were associated with elevated arterial superoxide in the overall patient population, an effect that was driven by nondiabetics. This effect was primarily due to eNOS uncoupling via reduced vascular BH4 (tetrahydrobiopterin) bioavailability. There was no significant impact of Lp(a) variability on vascular NOX–derived superoxide (*P*=0.13). RNA sequencing of arterial tissue revealed dysregulation of nitrosative and inflammatory signaling in high Lp(a) patients although there was no association with systemic biomarkers of inflammation (ie, hsCRP [high-sensitivity C-reactive protein]; *P*=0.82) or oxidative stress (ie, malondialdehyde; *P*=0.61). Finally, both LPA GRS (hazard ratio, 3.615 [95% CI, 1.044–12.515]; *P*=0.043) and high plasma Lp(a) (hazard ratio, 3.286 [95% CI, 1.003–10.767]; *P*=0.049) were associated with elevated risk for cardiac mortality. This association was vascular superoxide-dependent, implying that redox-sensitive inflammatory signaling may be a link between Lp(a) and cardiovascular risk. All the above associations were independent of plasma ApoB (apolipoprotein-B).

**CONCLUSIONS:**

This study demonstrates for the first time that a genetically determined increase in plasma Lp(a) results in dysregulated vascular redox/nitrosative signaling in patients with atherosclerosis.

**Highlights**

- Plasma Lp(a) (lipoprotein[a]) levels have an established association with cardiovascular disease and are suspected to be a consequence of the vascular inflammation that characterizes patients with coronary artery disease, yet the mechanisms underlying this association are not well understood.
- Owing to the fact that Lp(a) levels are largely genetically determined, we have used genetic tools to show that Lp(a) impacts vascular redox signaling associated with eNOS (endothelial nitric oxide synthase) uncoupling in an ApoB (apolipoprotein-B)–independent manner, and this may drive, at least partly, the previously described Lp(a)-driven risk for adverse cardiovascular outcomes.
- This study illustrates a potential key mechanism through which Lp(a) increases cardiovascular risk and provides a rationale for further mechanistic investigations incorporating the novel Lp(a)-lowering therapies that are entering clinical practice.

**See accompanying editorial by Zubiran and Remaley**

Coronary artery disease (CAD) is a dominant cause of global morbidity and mortality.^1^ Over the last few decades, preventive cardiovascular medicine has focused on controlling the levels of ApoB (apolipoprotein-B) containing molecules such as low-density lipoprotein cholesterol (LDL-C), originally with statins and recently with anti-PCSK9 (proprotein convertase subtilisin/kexin type 9) targeting.^2,3^ Yet, even with the reduction of plasma LDL-C, a residual CAD risk remains, which may be partly driven by inflammation, as shown in the CANTOS (Canakinumab Anti-Inflammatory Thrombosis Outcome Study) and LoDoCo (Low-Dose Colchicine) trials.^4,5^

Lp(a) (lipoprotein[a]) is a polymorphic lipoprotein particle that contains an ApoB molecule, covalently linked to the ApoB glycoprotein.^6^ Lp(a) has emerged as a new CAD risk factor, and novel therapeutic strategies incorporating RNA-interference and antisense oligonucleotides have been shown to reduce circulating Lp(a) although their impact on cardiovascular risk is awaited from ongoing clinical trials.^7–11^ Importantly, over 90% of the variation in plasma Lp(a) is genetically determined, largely through variants in or near the *LPA* gene such as the Kringle-IV repeat polymorphisms, which affect the size of the ApoB molecule and, thus, the size of the overall Lp(a) isoform.^6,12^ Carriers of smaller ApoB isoforms exhibit median plasma Lp(a) concentrations >5× higher than those with larger isoforms, as illustrated in the PROCARDIS study (Precocious Coronary Artery Disease Study).^8,12,13^ Mendelian randomization studies support the case for a causal association between Lp(a) and CAD, yet the mechanisms underlying this relationship are still not completely understood.^9^

Lp(a) also acts as a major carrier of oxidized phospholipids (ox-PLs), stimulating vascular inflammation and leukocyte extravasation.^14–17^ Other known ox-PL carriers (such as ox-LDL-C [oxidized low-density lipoprotein cholesterol]) preferentially donate ox-PLs to Lp(a), with plasma ox-PL and Lp(a) levels increasing proportionately in humans.^18^ The ox-PL content in Lp(a) molecules seems to derive from both lipid and nonlipid extractable pools, suggesting that ox-PLs can be found in the lipid phase of the Lp(a) molecule and covalently bound to the ApoB component.^18^ The difference in the ease of transferability of ox-PLs in the lipid phase of Lp(a) and those bound to ApoB could account for the mechanism through which Lp(a) acts as a major ox-PL carrier, yet further work investigating the modification of ox-PL from all known sources is needed to confirm this hypothesis.^18^

In addition, Lp(a) levels share interactions with systemic inflammatory biomarkers such as IL (interleukin) 6 and hsCRP (high-sensitivity C-reactive protein); IL6 inhibition reduces Lp(a) levels by ≈40%, while background hsCRP interacts with Lp(a)-driven cardiovascular risk.^9,19–22^ This suggests that high Lp(a) levels may increase as a result of systemic inflammation that characterizes patients with CAD and may be a rational therapeutic target in cardiovascular prevention. Yet, this relationship has been questioned by a more recent study of a larger sample size, showing an hsCRP-independent effect of Lp(a) on cardiovascular risk.^23^ Furthermore, the mechanistic links between Lp(a) and inflammation remain obscure. Inflammatory redox signaling, in particular, has been revealed as a key downstream driver of vascular inflammation, but its relationship with Lp(a) is unknown.

As a result, we aimed to explore the impact of Lp(a) on arterial superoxide (O_2_^.−^) and inflammatory redox signaling in humans, with the objective to better understand the molecular mechanisms behind such an association. Furthermore, we explored the impact of these observations on clinical outcomes in patients with CAD.

## Methods

Supporting data are available from the corresponding author upon reasonable request.

### Study Population

**Figure 1.**
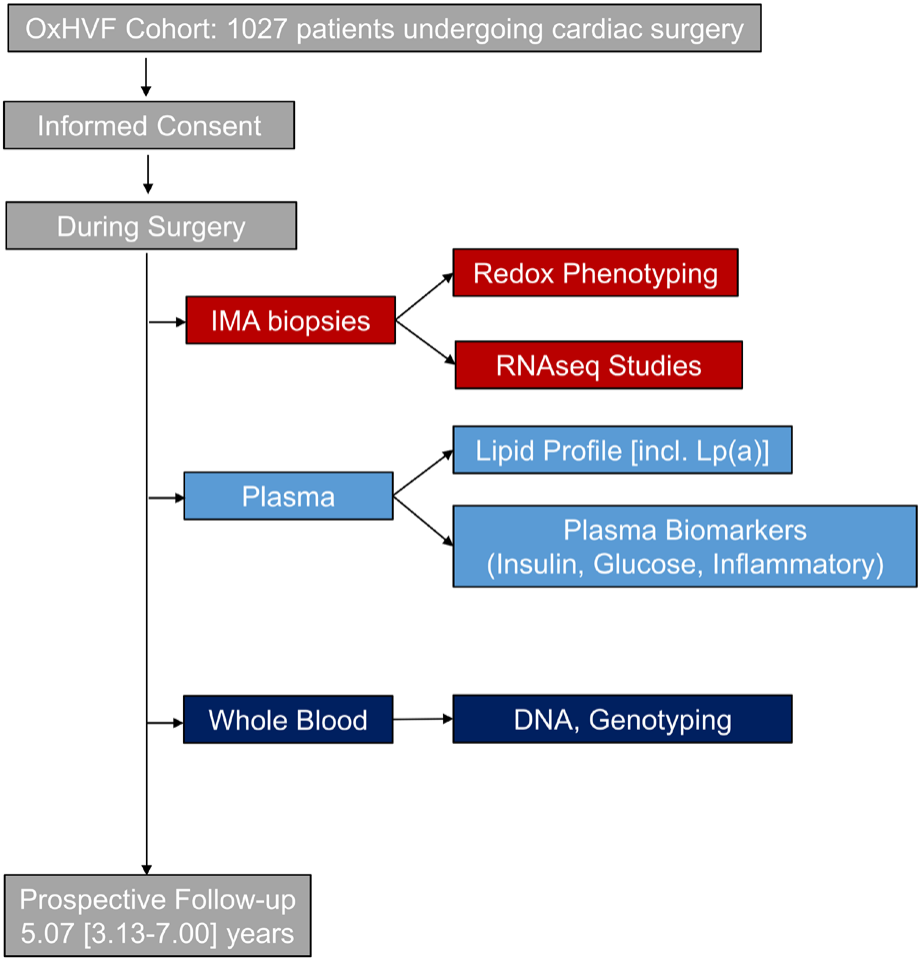
**Study design.** Schematic diagram depicting the sequence of tissue collection and processing, experimentation, and data collection. Lp(a) indicates lipoprotein(a); OxHVF, Oxford Cohort for Heart, Vessels, and Fat; and RNAseq, RNA sequencing.

The study population comprised 1027 patients with advanced CAD undergoing cardiac surgery at Oxford University Hospitals NHS (National Health Service) Foundation Trust, United Kingdom. All patients were recruited under the OxHVF (Oxford Cohort for Heart, Vessels, and Fat) programme (www.oxhvf.com; REC: 11/SC/0140). The study protocol was in accordance with the Declaration of Helsinki, and all patients provided written informed consent prior to enrollment. Figure 1 illustrates the study design. The CONSORT (Consolidated Standards of Reporting Trials) diagram for this study is presented in Figure S1. Further details can be found in the Supplemental Material.

### Clinical Phenotyping and Risk Factor Definitions

In the OxHVF study, the case report forms were completed at the time of patient recruitment, before the patients underwent cardiac surgery. Briefly, the study investigator took a focused medical history (including current medications) from the study participants prior to surgery. This information was then complemented using phenotypic data (including measurement of blood pressure, lipid profile, and HbA1c [hemoglobin A1C]) measured prior to surgery as recorded on electronic medical records. Any historical recorded diagnoses (based on *International Classification of Diseases* coding) from general practitioner records, referral letters, and electronic hospital records were also documented. The Primary and Secondary International Classification of Diseases, *Tenth Revision* codes for each patient’s encounter with the NHS were recorded from the NHS Digital databases, and they were used to identify new diagnoses from past history. These recorded data were then reviewed by an adjudication committee of 3 clinicians (adjudication team) to finalize the diagnostic classification of all information finalized in the study record. The clinical adjudication team followed the respective European Society of Cardiology guidelines for the diagnosis of hypercholesterolemia and hypertension, and the ADA (American Diabetes Association) guidelines for diabetes.^24–26^

### Blood Sampling and Circulating Biomarker Measurements

Immediately prior to surgery, venous blood was collected at the same time preheparinization and processed as previously described.^27^ Blood samples were stored at −80 °C until assayed. Plasma Lp(a) was measured using the Randox Lp(a) Assay (LP2757; Randox Laboratories Ltd) and reported in mg/dL. Plasma ApoB was measured using the Randox ApoB Assay (LP3839; Randox Laboratories Ltd). Lipid profile was measured as part of clinical care using an Architect analyzer (Abbott Laboratories). Further details are described in Supplemental Material.

### Genotyping and Genetic Analyses

**Figure 2.**
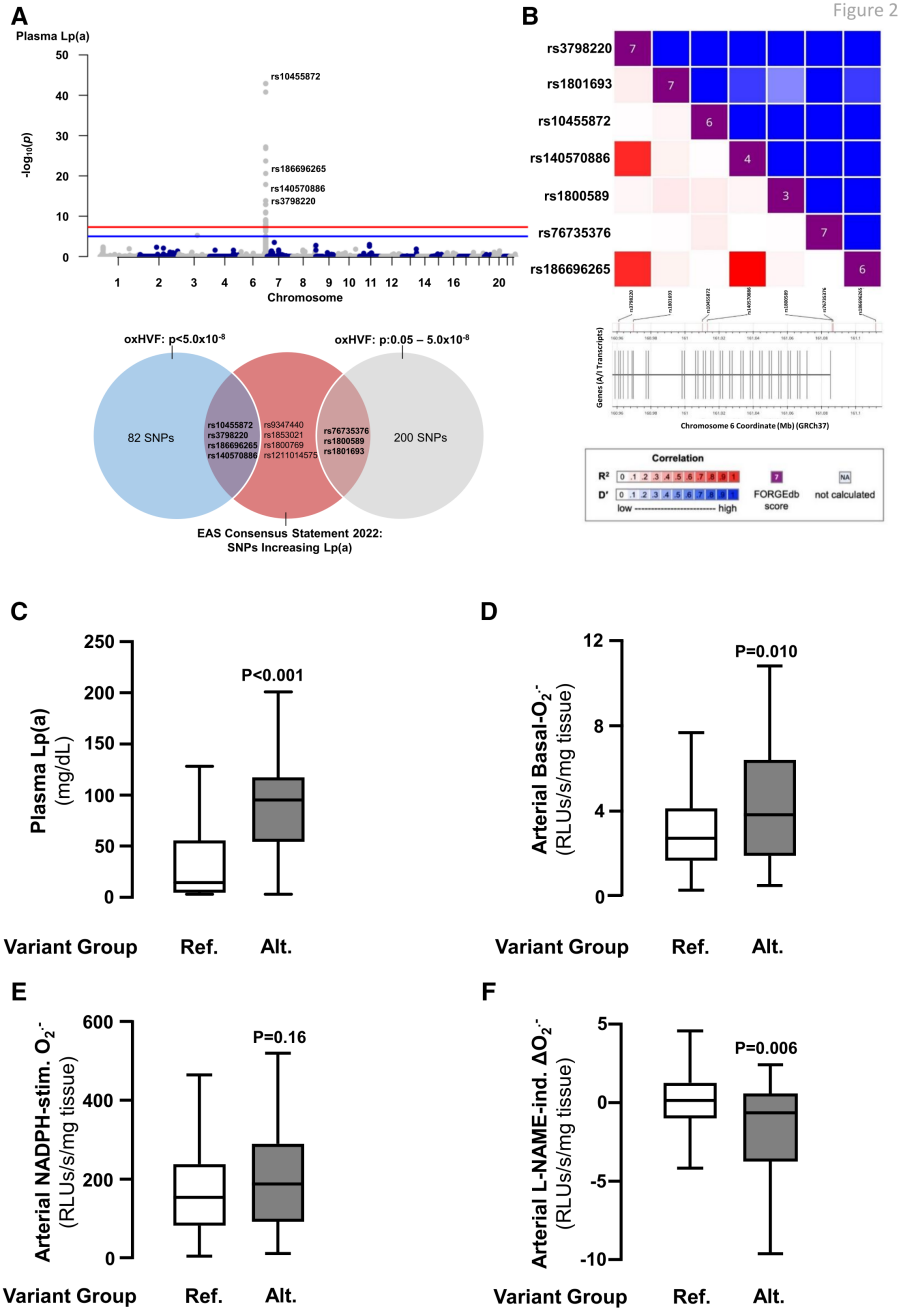
**The *LPA* genetic risk score (LPA GRS) is associated with increased arterial superoxide (O_2_^.−^) production. A**, Seven single nucleotide polymorphisms (SNPs) were included in the LPA GRS. The red line indicates genome-wide significance following correction for multiple testing using the false discovery rate method. **B**, The linkage disequilibrium (LD) statistics for these variants are arranged by genomic coordinates on the x and y axes, creating a square plot that indicates R^2^ below (in red), and D’ above (in blue), the FORGEdb score for each variant (in purple). Patients in the alternative LPA GRS variant group had significantly increased plasma Lp(a) (lipoprotein[a]) levels (n=666; **C**) compared with patients in the reference variant group. Compared with those in the reference LPA GRS variant group, patients in the alternative variant group had significantly increased arterial basal-O_2_^.−^ production (n=302; **D**). Furthermore, despite no significant difference in NOX (NADPH oxidase)-O_2_^.−^ production (n=297; **E**), patients in the alternative vs reference LPA GRS variant group had significantly increased eNOS (endothelial nitric oxide synthase)–derived O_2_^.−^ production (n=267; **F**). Alt indicates alternative; Ind., induced; Med., medium; Ref., reference; and RLU, relative light unit.

DNA was extracted from whole blood using the QIAamp DNA Blood Midi Kit (51185; Qiagen) according to the manufacturer’s instructions. Genotyping of the study population was performed using the Affymetrix platform using the UK Biobank Axiom Array as previously described (Supplemental Material).^28^ The modified *LPA* genetic risk score (LPA GRS) was created using an unweighted summative model as previously described.^28,29^ Here, genome-wide association screening was performed for plasma Lp(a) identifying 82 single nucleotide polymorphisms (SNPs) above genome-wide significance that correlated with Lp(a) levels (Figure 2A). Four of these SNPs were among those identified by the European Atherosclerosis Society (EAS) 2022 consensus statement as clinically relevant to Lp(a) levels.^9^ Another 3 SNPs from the EAS list were also found within the OxHVF longlist with a significance threshold lowered to *P*<0.05.^9^ The 7 SNPs (rs10455872, rs3798220, rs186696265, rs76735376, rs1800589, rs140570886, and rs1801693) were then included in the LPA GRS, as shown in Figure 2. The LDL-C polygenic risk score was created using a weighted summative model as previously described (Supplemental Material).^30^

### Human Tissue Collection and Processing

Arterial biopsies of the internal mammary artery were harvested during surgery using a no touch technique and transferred to the laboratory within 30 minutes to commence processing and analysis. All sample collection and processing protocols were standardized with samples collected approximately at the same stage of the surgery for all patients (Supplemental Material).^27^

### Vascular Redox State Bioassays

Vascular O_2_^.−^ generation was measured on the fresh arterial samples using lucigenin (5 μmol/L)-enhanced chemiluminescence as we have previously described (Supplemental Material).^27,31,32^ The contribution of uncoupled eNOS (endothelial nitric oxide synthase) was evaluated by using the NOS inhibitor L-N^G^-nitro arginine methyl ester (L-NAME; O_2_^.−^ generation was measured before and after the addition of L-NAME to calculate the delta(O_2_^.−^) induced by L-NAME (ie, delta(O_2_^.−^)=[O_2_^.−^ after adding L-NAME]−[O_2_^.−^ before adding L-NAME]), while the contribution of NOXs (NADPH oxidases) was assessed by measuring the NADPH-stimulated O_2_^.−^ as well by using the pan-NOX inhibitor Vas2870, as previously described (Supplemental Material).^27,31,32^

### Oxidative Fluorescent Microscopy

To visualize vascular O_2_^.−^ production within the vascular wall, cryosections of optimal cutting temperature compound–embedded arterial biopsies were processed with O_2_^.−^ fluorescent indicator dihydroethidium and visualized using confocal microscopy as previously described (Supplemental Material).^33^

### Measurement of Plasma Biopterins

Plasma BH4 (tetrahydrobiopterin) and BH2 (dihydrobiopterin) levels were each determined separately using high-performance liquid chromatography followed by serial electrochemical and fluorescent detection as previously described (Supplemental Material).^31,34^ Tissue biopterin levels were expressed as nmol/L in plasma.

### RNA-Sequencing Analyses

Details of the RNA extraction, sequencing, and data processing of arterial samples are provided in the Supplemental Material. Transcriptomic data analyses were conducted as previously described.^35^

### Statistical Analyses

All sample sizes represent biological replicates (individual patients). The distribution of continuous variables was assessed for normality using the Kolmogorov-Smirnov test. Normally distributed variables are presented as mean±SEM and nonnormally distributed variables as median (interquartile range; whiskers [1.5×interquartile range]; outliers [<Q1–1.5×interquartile range or >Q3+1.5×interquartile range]). Correlation analyses were performed using the Pearson (for normally distributed variables) or Spearman rank (for nonnormally distributed variables) correlation coefficients. Comparisons of continuous variables between 2 groups were performed using the unpaired Student *t* test or the Mann-Whitney *U* test.

To test whether the association of arterial basal-O_2_^.−^ with the LPA GRS and plasma Lp(a) was independent of plasma ApoB and other clinical/demographic characteristics, we performed general linear regression analyses using arterial basal-O_2_^.−^ as a dependent variable and independent variables as stated. When interrogating the direction of effects in univariate associations, plasma Lp(a) was categorized into tertiles. Standardized betas (B) are presented for each covariate. To examine the prognostic value of Lp(a) levels or genetic variability for the primary outcome of cardiovascular mortality, we performed Cox regression survival analyses; hazard ratios (HRs; 95% CI) are presented after correction for relevant covariates. Parameter estimates were bootstrapped in cases where both the Levene test gave a significant result, and inspection of scatterplots suggested that the assumption of homoscedasticity of residuals had been violated. For the cis-Mendelian randomization (MR), 3 main instrumental variable assumptions were made: the instrumental variable was robustly associated with plasma Lp(a) levels (relevance), this association was not affected by confounding factors (independence), and the instrumental variable only affected a clinical outcome via the specified exposure (exclusion restriction).^36,37^ The relevance assumption was assessed by inspecting the F-statistic; if this was >10, the association was considered robust and weak instrument bias minimal. Violation of the third assumption was reduced by using cis-MR. MR-Egger regression was used as a sensitivity analysis as it is robust to violations of the exclusion restriction assumption.^38^

Power calculations were based on arterial basal-O_2_^.−^, and we estimated that with 100 patients, we could detect a difference of 0.67 in log(arterial basal-O_2_^.−^) between low versus medium/high plasma Lp(a) with 90% power, α=5%, and assuming an SD of 0.28. All statistical analyses were performed using SPSS, version 29.0 (IBM, United States). A *P* value of <0.050 was considered statistically significant. Adjustments for multiple comparisons were performed using the false discovery rate where appropriate.

## Results

The patient demographic characteristics are presented in Table 1. Initial analyses were conducted in the entire study population (n=1027) followed by subgroup analyses according to diabetic status. The median age was 67.05±0.3 years. The mean±SEM time between patient recruitment and surgery was 3.14±0.47 days, with 84.7% of patients undergoing surgery within 48 hours of recruitment. No patients experienced an interceding myocardial infarction event during the recruitment-surgery window; 79.1% (n=815) were nondiabetic, and 20.9% (n=215) of patients were diabetic. Comparing the nondiabetic and diabetic cohorts, there were statistically significant differences in Homeostatic Model Assessment for Insulin Resistance, hypertension, history of myocardial infarction, hypercholesterolemia (as well as serum cholesterol, LDL-C, HDL [high-density lipoprotein], and triglycerides), body mass index, waist-to-hip ratio, plasma ApoB, plasma Lp(a), antiplatelet medications, angiotensin-receptor blockers, statins, and calcium-channel blockers.

**Table 1.**
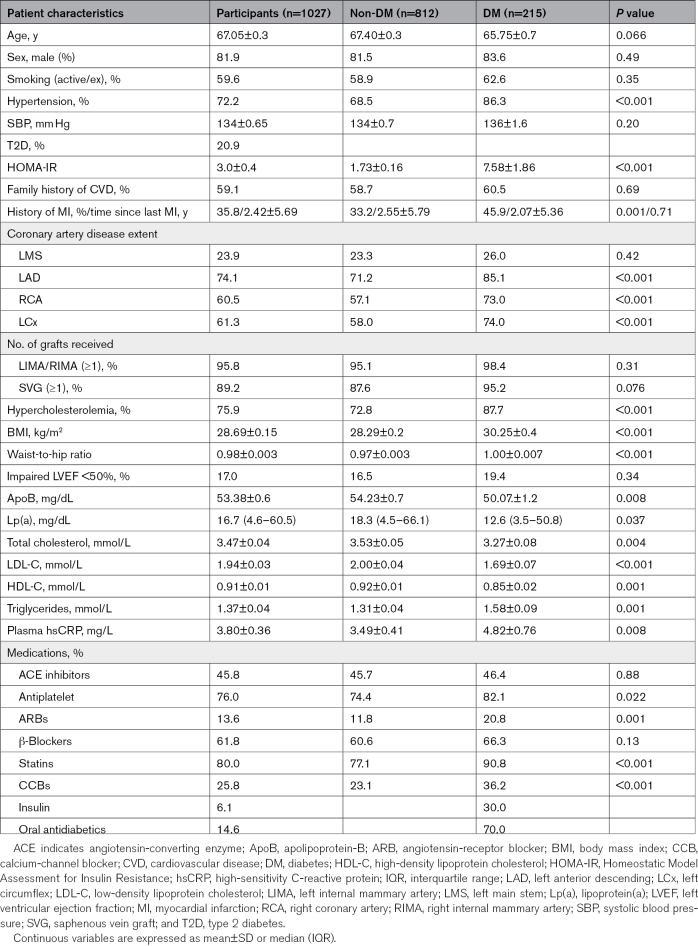
Demographic Characteristics of the Study Population

### Using Mendelian Randomization to Test Causality Between Lp(a) and Vascular Redox State

To construct a genetic signature for use as a Mendelian randomization tool, we first searched for externally identified genetic polymorphisms significantly associated with plasma Lp(a), which were then tested for their ability to capture Lp(a) levels in the study population. We identified 11 SNPs from the 2022 EAS consensus statement, which were proposed as determinants of Lp(a) levels.^9^ In the OxHVF cohort, 4 of these SNPs (rs10455872, rs3798220, rs186696265, and rs140570886) were found to be significantly associated with plasma Lp(a) levels, within a genome-wide significance threshold of *P*<5.0×10^−8^ (Figure 2A). Further 3 SNPs from the EAS consensus statement were also found to be correlated with high plasma Lp(a) levels in the study population at *P*<0.05 (rs76735376, rs1800589, and rs1801693).^9^ The remaining variants from the EAS consensus statement were not available in the OxHVF cohort so were not included. Subsequently, we confirmed that the alternative allele of each of these 7 SNPs was individually associated with higher levels of plasma Lp(a), as shown in Figure S2. We then conducted quantitative trait association tests and combined these 7 SNPs to construct a modified genetic signature of Lp(a) in this population with advanced CAD (Table S1).^9^ A modified *LPA* genetic risk score (LPA GRS) was then generated, in which patients with any alternative allele from ≥3 of these *LPA* SNPs (the alternative LPA GRS group) were compared with those with no alternative alleles in any of these *LPA* SNPs (the LPA GRS reference group). Then, we conducted linkage disequilibrium analyses using the National Institutes of Health LDmatrix tool (accessed at https://ldlink.nih.gov/?tab=ldmatrixhttps://LDL-Cink.nih.gov/?tab=ldmatrix) and confirmed a high degree of linkage disequilibrium between the 7 selected SNPs according to D’ (Figure 2B). FORGEdb scores were also provided and indicate a moderate to a high degree of functional relevance for these SNPs (Figure 2B).^39^

We identified that patients in the alternative LPA GRS variant group had significantly higher plasma Lp(a) compared with those in the reference group (Figure 2C). In our assessment of the assumptions made of the instrumental variable in the cis-MR analysis, we confirmed that the risk of weak instrument bias was minimal, given that the F-statistic for the association between LPA GRS and plasma Lp(a) was >10 (F=35.454). This association was independent of confounding variables. Bias due to horizontal pleiotropy was likely minimal as cis-MR was used, and the intercept from MR-Egger regression was not significantly different from zero (*P*=0.819). This confirmed the validity of the genetic tool for testing causality between Lp(a) levels and any clinical phenotype.

To explore the impact of genetically determined Lp(a) variability on vascular redox state, we tested for associations between the LPA GRS variant groups and arterial O_2_^.−^, measured in fresh arterial biopsies. Indeed, patients in the alternative LPA GRS variant group had significantly higher basal arterial O_2_^.−^ (Figure 2D). To explore the underlying enzymatic sources contributing to this finding, we focused on NOXs and uncoupled eNOS. There was no significant difference in arterial NADPH-stimulated O_2_^.−^ (Figure 2E), but there was a significantly greater L-NAME-induced delta(O_2_^.−^) in patients in the alternative LPA GRS variant group (Figure 2F) compared with the reference LPA GRS genetic variant group, implying that genetically determined variability of *LPA* may affect eNOS uncoupling. Interestingly, the association between the genetic variability of the LPA GRS and vascular redox state was driven by patients without diabetes (Figure S3A through S3C), while the associations lost significance in patients with diabetes (Figure S3D through S3F). Of note, there was no direct association between the LPA GRS and diabetic status (χ^2^=0.233; *P*=0.629), whereas the LPA GRS was strongly associated with plasma Lp(a) in both patients without diabetes and patients with diabetes (Figure S4A and S4D).

### Plasma Lp(a) and Arterial Redox State

**Figure 3.**
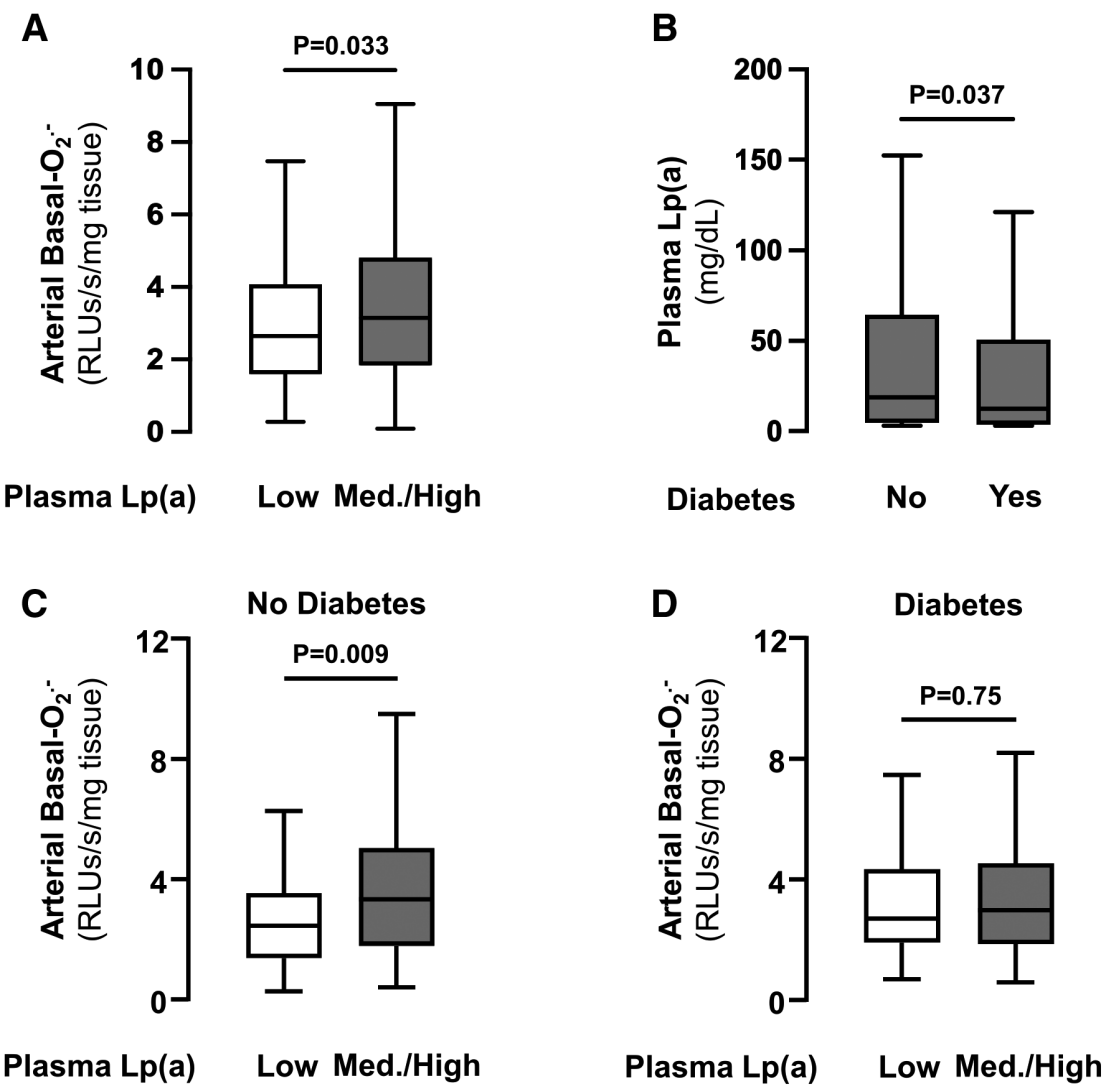
**Lp(a) (lipoprotein[a]) increases arterial superoxide (O_2_^.−^) production in nondiabetics but not in diabetics.** Patients with medium/high levels of Lp(a) had significantly increased arterial basal-O_2_^.−^ production compared with those with low plasma Lp(a) levels in the entire study population (n=411; **A**). Patients with diabetes had significantly lower plasma Lp(a) compared with nondiabetics (n=947; **B**). Patients without diabetes (n=147; **C**) with medium/high levels of Lp(a) had significantly increased arterial basal-O_2_^.−^ production compared with those with low plasma Lp(a) levels. In patients with diabetes, there was no significant difference in arterial basal-O_2_^.−^ production between those with medium/high vs low plasma Lp(a) levels (n=149; **D**). Med indicates medium; and RLU, relative light unit.

We then sought to explore the relationship between plasma Lp(a) and vascular redox state. Patients with high plasma Lp(a) levels (top tertile versus bottom 2 tertiles) had significantly elevated arterial O_2_^.−^ (Figure 3A). Furthermore, patients with diabetes had significantly lower plasma levels of Lp(a) (Figure 3B), and the association between plasma Lp(a) and arterial O_2_^.−^ was driven by patients without diabetes (Figure 3C and 3D). A moderation analysis further confirmed that the interaction between plasma Lp(a) levels and arterial O_2_^.−^ production was moderated by diabetic status (b, −1.58 [95% CI, −3.15 to −0.01]; *P*=0.048). Given the confounding interaction of diabetes status with Lp(a) levels, subsequent analyses to understand the mechanisms behind Lp(a) and vascular redox state were focused on patients without diabetes.

**Figure 4.**
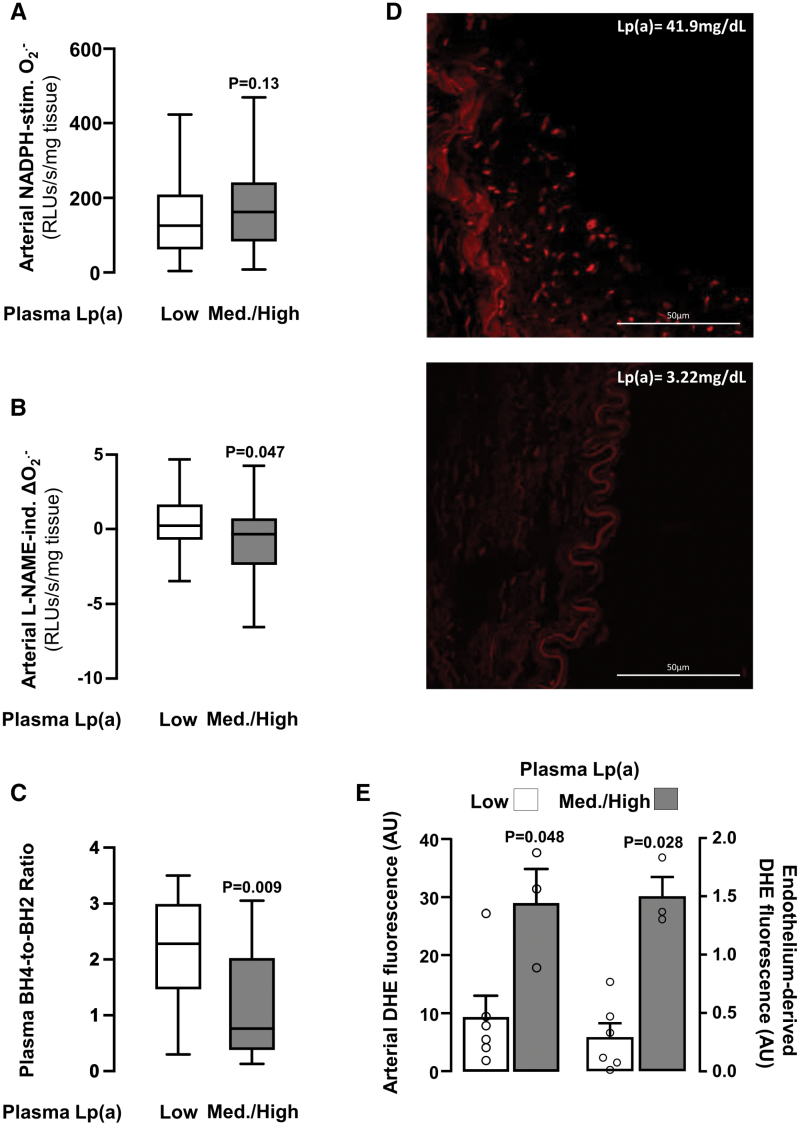
**Lp(a) (lipoprotein[a]) is associated with an increase in eNOS (endothelial nitric oxide synthase)–derived arterial superoxide (O_2_^.−^) production.** Despite no significant difference in NOX-derived O_2_^.−^ production (n=143; **A**), patients without diabetes with medium/high vs low plasma Lp(a) levels had significantly increased eNOS-derived (n=130; **B**) O_2_^.−^ production. Furthermore, patients with medium/high vs low plasma Lp(a) levels had a significantly reduced tetrahydrobiopterin (BH4)-to-dihydrobiopterin (BH2) ratio (n=40; **C**). Dihydroethidium (DHE) staining images for in situ visualization (**D**) and quantification (n=9; **E**) of basal arterial O_2_^.−^ production in internal mammary artery (IMA) segments from patients with high and low plasma Lp(a). Ind. indicates induced; and Med., medium.

We then explored the contribution of NOXs and uncoupled eNOS in this association. Indeed, while there was no significant difference in NADPH-stimulated O_2_^.−^ between patients without diabetes with high versus low plasma Lp(a) (Figure 4A), high plasma Lp(a) was related to significantly higher levels of arterial L-NAME-induced delta(O_2_^.−^) compared with those with low plasma Lp(a) (Figure 4B). Importantly, patients with high Lp(a) levels had significantly lower arterial BH4 bioavailability, as defined by the ratio of plasma BH4 to its oxidation product BH2 (Figure 4C), providing a mechanistic explanation of the association between Lp(a) and eNOS uncoupling in the human arterial wall. These findings were confirmed by visualizing arterial O_2_^.−^ production in human arterial wall using dihydroethidium staining, confirming that high plasma Lp(a) levels are related to a global increase of arterial O_2_^.−^, including higher endothelium-derived O_2_^.−^. This further supports the notion that Lp(a) may trigger eNOS uncoupling in the human arterial wall (Figure 4D and 4E).

Given the association between plasma Lp(a) and ApoB, we then sought to explore whether ApoB could be a confounder of the association between Lp(a) and vascular O_2_^.−^. Patients without diabetes, but not diabetics, with high plasma Lp(a) had significantly higher levels of plasma ApoB (Figure S4B and S4E). This was corroborated on a genetic level where patients without diabetes, but not diabetics, in the alternative LPA GRS variant group had higher plasma ApoB compared with those in the reference group (Figure S4C and S4F). Importantly, the association of plasma Lp(a) and the LPA GRS with arterial O_2_^.−^ was independent from ApoB levels and an LDL-C polygenic risk score (Table 2). Furthermore, there was no significant association between circulating hsCRP or MDA (malondialdehyde) and plasma Lp(a) (Figure S5A and S5B) or the LPA GRS (Figure S5C and S5D), as well as no significant association between arterial L-NAME-induced delta(O_2_^.−^) and circulating hsCRP (Figure S5E). Finally, there was no significant association between plasma Lp(a) and the arterial expression of IL6, IL1b, and NLRP3 (NLR family pyrin domain-containing 3) nor was there any significant association between arterial L-NAME-induced delta(O_2_^.−^) and circulating IL6, arterial IL1B expression, or arterial NLRP3 expression (Figure S6). This suggests that the association between plasma Lp(a) and vascular O_2_^.−^ is distinct and not appreciably affected by the aforementioned inflammatory pathways.

**Table 2.**
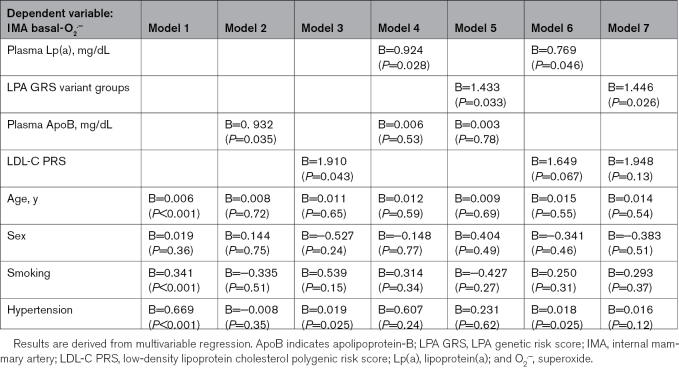
Associations Between Lp(a) and Arterial Basal-O_2_^.−^ in Patients Without Diabetes

### Lp(a) and Arterial Transcriptomic Profile

**Figure 5.**
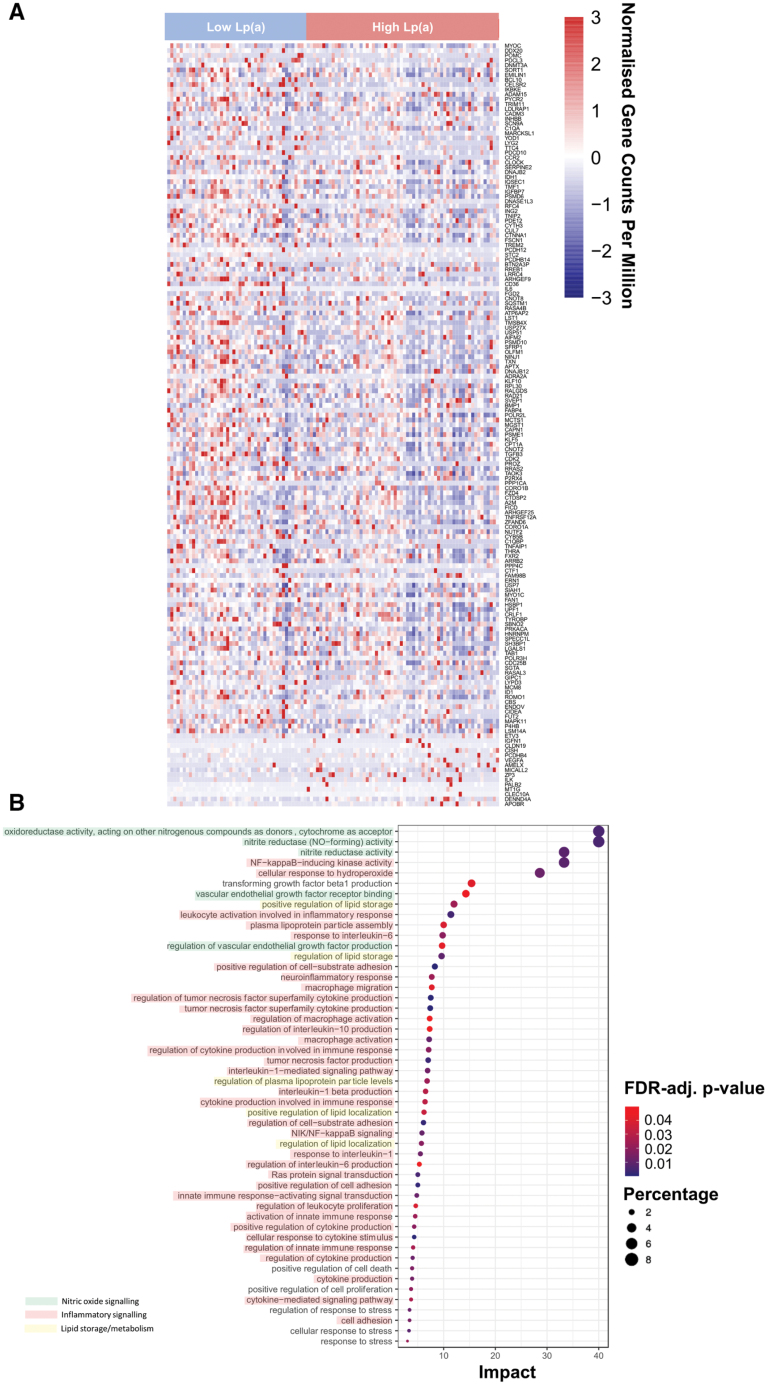
**The pathways linking Lp(a) (lipoprotein[a]) and the dysregulated vascular redox state.** Heatmaps of the most significantly differentially expressed genes (DEGs; following correction for multiple testing using the false discovery rate [FDR] method) in patients without diabetes with high vs low plasma Lp(a) levels (n=108; **A**). The top 50 gene ontology pathways were ranked by impact from a gene set enrichment analysis of these DEGs, which identified significantly enriched pathways for all patients with high vs low plasma Lp(a) levels (n=108; **B**). The node color is based on the FDR-adjusted (FDR-adj.) *P* value, and the node radius is determined on the basis of pathway impact values. High-impact pathways relevant to nitric oxide signaling/endothelial function are highlighted in green, those relevant to inflammatory signaling are highlighted in red, and those relevant to lipid storage/metabolism are highlighted in yellow.

To further explore the mechanisms underlying the link between Lp(a) and a dysregulated vascular redox state, we investigated the effect of plasma Lp(a) on the transcriptomic profile of 208 human internal mammary arterial biopsies from patients without diabetes and patients with diabetes using RNA sequencing. High Lp(a) was defined as plasma Lp(a) at the highest tertile and low Lp(a) as patients in the lowest tertile of plasma Lp(a). Between patients without diabetes with high versus low plasma Lp(a), we observed 157 significantly differentially expressed genes (Figure 5A). We then performed a gene set enrichment analysis of these differentially expressed genes, identifying the top 50 significantly enriched gene ontology pathways between high versus low plasma Lp(a) (Figure 5B; Table S2). Among these enriched pathways, the vast majority of these enriched pathways were related to nitric oxide signaling/endothelial function and vascular inflammatory responses/redox signaling, with a small number of pathways relevant to lipid storage and lipoprotein metabolism. Indeed, the top enriched Gene Ontology terms linked to oxidoreductase/nitrite reductase activity and cellular response to oxidative stress contained the 3 antioxidant genes *CBS*, *CYB5B*, and *MGST1* that were found downregulated in the high plasma Lp(a) group.^40–42^ We validated these findings by comparing the transcriptomic profile of patients without diabetes with high versus low plasma Lp(a) to that of 57 patients without diabetes with high versus low arterial L-NAME-induced delta(O_2_^.−^). High L-NAME-induced delta(O_2_^.−^) was defined as being at the highest tertile and low L-NAME-induced delta(O_2_^.−^) as being at the lowest tertile of this readout in the study population. We observed 334 differentially expressed genes between patients without diabetes with high versus low arterial L-NAME-induced delta(O_2_^.−^). A further gene set enrichment analysis of these differentially expressed genes identified 16 significantly enriched gene ontology pathways between high and low L-NAME-induced delta(O_2_^.−^), which were also significantly enriched between patients with high versus low plasma Lp(a) (Figure S7). Most of these commonly enriched pathways are related to vascular inflammatory responses with a small number relevant to cell death (Table S3). These findings further support the role of eNOS-dependent redox dysregulation related to overwhelming activation of inflammatory pathways in the human arterial wall in patients with high Lp(a). Results for RNA sequencing analyses in all patients (diabetics and nondiabetics) in the study are shown in Figure S8 and Tables S4 and S5 (there were no common significantly enriched pathways between L-NAME-induced delta(O_2_^.−^) and Lp(a) in all patients).

### Lp(a) Is Causally Associated With Cardiovascular Death Independent of ApoB

**Figure 6.**
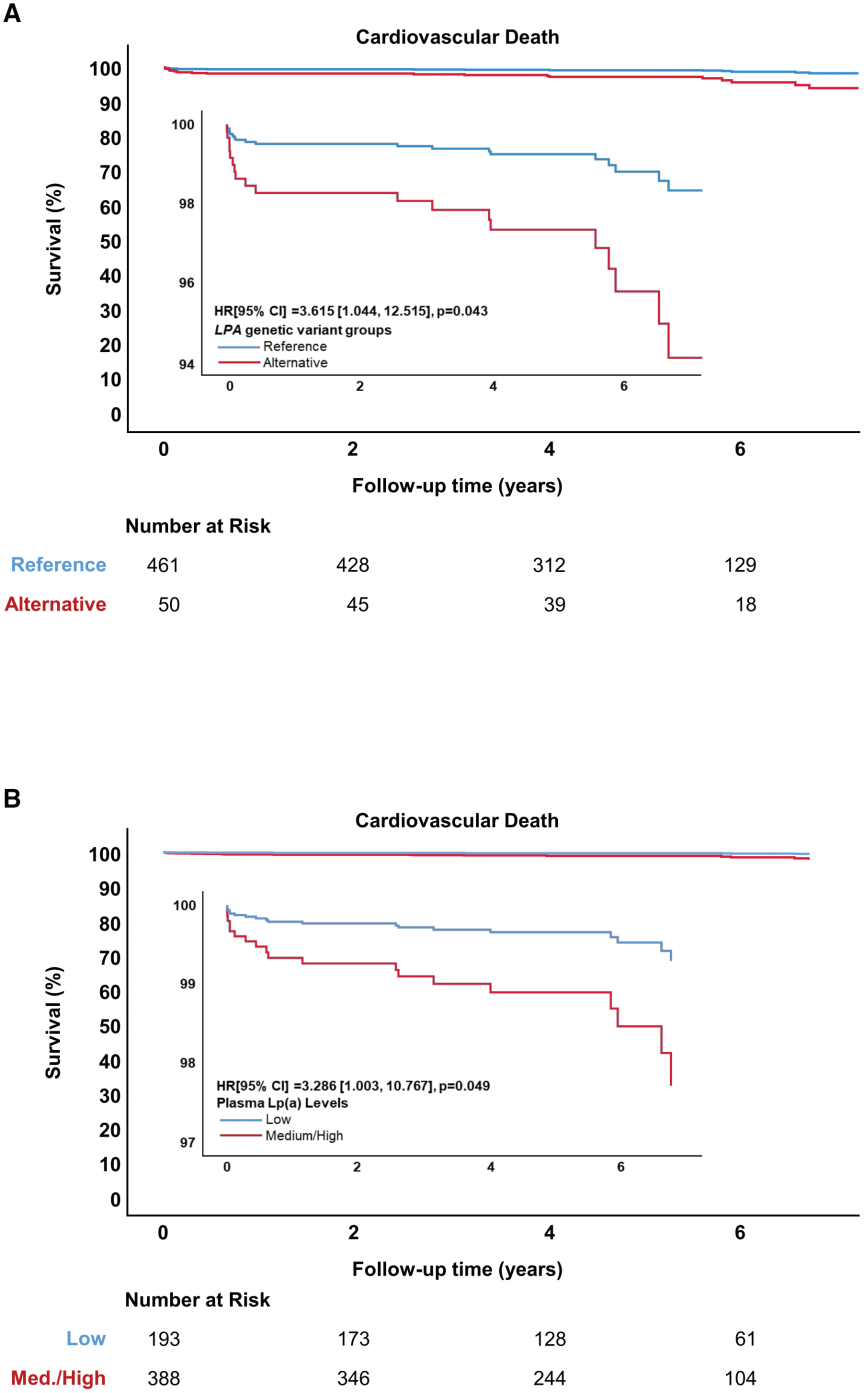
**Lp(a) (lipoprotein[a]) is causally associated with an increase in the observed risk of cardiovascular death.** In the whole study cohort, patients in the alternative *LPA* genetic risk score (LPA GRS) variant group had a significantly increased risk of cardiovascular death compared with those in the reference variant group (n=20 events; **A**). Similarly, patients with medium/high levels of plasma Lp(a) had a significantly increased risk of cardiovascular death compared with those with low levels (n=19 events; **B**). Cox regression models were adjusted for age, sex, hypertension, smoking, body mass index (BMI), diabetic status, family history of cardiovascular disease, history of myocardial infarction, urgency of surgery, and plasma ApoB (apolipoprotein-B). HR indicates hazard ratio.

Given the link between Lp(a), vascular redox signaling, and O_2_^.−^ production, we used Mendelian randomization to assess the causal effect of Lp(a) on prospective clinical outcomes. We found that patients in the alternative LPA GRS variant group had a significantly higher risk of cardiovascular death (HR, 3.62 [95% CI, 1.04–12.52]; *P*=0.04) compared with those in the reference group, independent of plasma ApoB (Figure 6A). In addition, when the modified LPA GRS was analyzed as a continuous variable in patients with an alternative allele from >1 of the included genetic variants, it was also associated with a significant increase in the risk of cardiovascular death (adjusted HR, 2.57 [95% CI, 1.32–5.02]; *P*=0.005; n=306; n=14; per SD). Correspondingly, patients with medium/high plasma Lp(a) also had a significantly higher risk of cardiovascular death (HR, 3.286 [95% CI, 1.003–10.767]; *P*=0.049) compared with those with low plasma Lp(a), again independent of plasma ApoB (Figure 6B). This confirms that Lp(a) is associated with increased cardiovascular mortality in an ApoB-independent manner, confirming prior observations by Trinder et al.^43^ Furthermore, when the cox regression models were additionally corrected for arterial O_2_^.−^ production, both the models for the *LPA* genetic signature (HR, 2.053 [95% CI, 0.180–23.428]; *P*=0.563) and plasma Lp(a) (HR, 1.700 [95% CI, 0.325–8.885]; *P*=0.529) lost significance. Importantly, the collinearity assumption was not violated owing to the correlation between plasma Lp(a) and arterial O_2_^.−^ production (ρ=0.124; *P*=0.030). This provides a proof of concept for the interaction of Lp(a) with arterial redox state in influencing clinical adverse events, supporting a causal relationship between Lp(a) levels and arterial oxidative stress. This suggests a key role for vascular O_2_^.−^ as a mediator of Lp(a)-driven cardiovascular events.

## Discussion

This study demonstrates, for the first time, that circulating Lp(a) is associated with arterial O_2_^.−^ production independently from ApoB and other cardiovascular disease risk factors, primarily in individuals without diabetes. Indeed, high Lp(a) levels are associated with vascular redox dysregulation, driven by greater eNOS uncoupling through the oxidation of eNOS-cofactor BH4. Finally, we use genetic tools to support a causal association between plasma Lp(a) and arterial redox signaling, which results in increased risk of cardiovascular mortality in patients with advanced atherosclerosis.

Despite existing evidence for a causal link between Lp(a) and atherosclerosis, the underlying mechanisms are incompletely understood.^44,45^ Under the oxidation hypothesis of atherogenesis, the initiating element of atherogenesis is the accumulation of oxidized LDL-C in the arterial wall that leads to foam cell formation, triggering atherosclerotic plaque development.^46–48^ Oxidative stress is a key mechanism in atherogenesis, and ox-LDL has been shown to increase O_2_^.−^ production by inducing eNOS uncoupling.^49,50^

Less is known about the relevance of Lp(a) in this process. Exposure to in vitro oxidized Lp(a) has been shown to result in the suppression of endothelium-dependent vasodilation, yet, up until now, there has been no evidence to suggest that Lp(a) is directly involved in arterial eNOS–dependent O_2_^.−^ production.^51^ Importantly, our results demonstrate that Lp(a)-associated arterial O_2_^.−^ production is independent of ApoB, showing that Lp(a) is not merely a conduit for the deleterious effects of oxidized LDL-C in the arterial wall but directly promotes the development of vascular oxidative stress. Furthermore, using Mendelian randomization, we have shown that the causal association between Lp(a) and an increased risk of cardiovascular mortality is also ApoB-independent and occurs through increasing arterial O_2_^.−^ production.

We have previously demonstrated that, in patients with advanced atherosclerosis, dysregulated postreceptor insulin signaling in the vascular wall is central to the production of excess ROS (reactive oxygen species), and the treatment of patients with DPP4 (dipeptidyl peptidase-4) inhibitors mitigates the insulin-induced development of vascular oxidative stress in an AMPK (adenosine monophosphate-activated protein kinase)–dependent manner.^27^ In light of this, we explored the relationship between Lp(a) and insulin sensitivity, given the established association between low plasma Lp(a) (20th percentile) and increased incident risk of type 2 diabetes.^9,52^ Our results demonstrate that patients with type 2 diabetes in our cohort had significantly lower plasma Lp(a) levels. There is no clear mechanistic consensus for the observed inverse association between Lp(a) levels and the risk of type 2 diabetes.^53^ One possible explanation is the increased glycation of ApoB in diabetics, who have higher molecular weight ApoB isoforms compared with genotype-matched individuals without diabetes.^54,55^ This could result in lower Lp(a) levels as glycated and nonglycated Lp(a) are catabolized at different rates.^53,56^ Another contributor could be the posttranscriptional suppression of ApoB synthesis in the presence of high insulin concentrations.^57^ Insulin also directly reduces ApoB-100 concentrations, which, together with the effects of insulin on increasing the production of triglyceride-rich VLDL (very-low-density lipoprotein) isoforms, could limit the availability of ApoB-100 isoforms to covalently bind to ApoB as part of the synthesis of Lp(a) molecules.^58,59^ This might also explain the inverse association observed between plasma Lp(a) and triglyceride levels.^59^

In addition, our finding that Lp(a) is associated with eNOS-derived arterial O_2_^.−^ production in nondiabetic, but not in patients with diabetes is indicative of the potential involvement of local vascular insulin signaling in this process, as insulin resistant patients with low plasma Lp(a) levels are likely to demonstrate increased arterial O_2_^.−^ production through additional, Lp(a)-independent, mechanisms. This suggests that, in our cohort, patients with diabetes (who were more likely to exhibit severe CAD) had plasma Lp(a) levels that more closely resembled the underlying population distribution, whereas patients without diabetes were more likely to have high Lp(a) as a driver of CAD. This is in line with data from Patel et al^60^ who showed that while elevated Lp(a) is a risk factor for CAD in both patients with diabetes and patients without diabetes, the presence of diabetes significantly attenuates this risk. Given that the association between plasma Lp(a) and arterial O_2_^.−^ production is independent of plasma ApoB in our study, the different interaction between the diabetic versus nondiabetic human arteries and Lp(a) levels could be due to different responsiveness of the vascular wall to plasma Lp(a) in patients with diabetes. Nevertheless, the pleiotropic effect of diabetes medications or insulin therapy on the Lp(a)-redox relationship in patients with diabetes cannot be excluded.

From a mechanistic standpoint, oxidized(a) has previously been shown in human endothelial cells to induce autophagy through an ROS-dependent AMPK-mTOR (mammalian target of rapamycin) pathway.^61^ Nevertheless, how Lp(a) might induce arterial oxidative stress has not been fully investigated. In atherosclerosis, excess vascular O_2_^.−^ oxidizes eNOS-cofactor BH4 to BH2, which results to eNOS uncoupling that shifts the flow of electrons on to molecular oxygen,^62–64^ turning this enzyme from a producer of NO to a source of O_2_^.−^, which initiates a self-reinforcing cycle that leads to further endothelial dysfunction, oxidizes remaining NO into peroxynitrite, and directly damages cellular components such as nuclear and mitochondrial DNA.^62,65,66^ Our results corroborate this by demonstrating that Lp(a) is associated with reduced vascular BH4 bioavailability and also with an increase in eNOS uncoupling, without any changes in NOX-derived arterial O_2_^.−^ in patients with atherosclerosis. Furthermore, at a transcriptomic level, we demonstrate that high plasma Lp(a) is associated with the differential expression of genes involved in regulating vascular redox state, and the ApoB-independent, eNOS-derived arterial O_2_^.−^ production seen in patients with elevated Lp(a) is also associated with dysregulated lipid storage in the vascular wall. Importantly, high plasma Lp(a) is related to an overwhelming activation of inflammatory pathways in the human arterial wall, despite the lack of any association between plasma Lp(a) and systemic markers of either inflammation (eg, hsCRP) or oxidative stress (eg, MDA), suggesting that the effects of Lp(a) on redox state and inflammatory signaling are happening at a local level within the vascular wall, rather than systemically. This is in line with previous findings that observed no association between plasma hsCRP and plasma Lp(a), reinforcing the premise of our work that the effects of Lp(a) are primarily on local vascular redox state, and the impact on inflammatory signaling within the vascular wall is downstream to that. Indeed, plasma Lp(a) was not related to the expression of most of the mainstream inflammatory genes but rather with vascular endothelial activation, which is unlikely to drive systemic levels of inflammatory biomarkers such as hsCRP or plasma cytokine levels. Although vascular inflammation may still be a rational therapeutic target in patients with high Lp(a), further research is needed to identify the exact inflammatory pathways to target in these patients.

Finally, high Lp(a) has been associated with a high risk for cardiovascular mortality. However, its role as a clinical predictor in patients with advanced CAD remains unknown. This study is the first to demonstrate that plasma Lp(a) levels are associated with higher risk of cardiovascular death in patients with advanced CAD undergoing cardiac surgery, and this effect was found to be dependent on vascular O_2_^.−^, as measured in arterial biopsies at the time of surgery, highlighting the key role of vascular redox dysregulation as a link between plasma Lp(a) levels and cardiovascular risk.

Owing to the complexity of isolating a standard human Lp(a) isoform, we could not perform ex vivo incubations to corroborate the mechanistic effects of Lp(a) on the human vascular wall, so investigations for causality of the associations are limited to the Mendelian randomization experiments. Furthermore, no measurements of plasma ox-PL levels were performed, and this is a limitation of the study. In addition, as we were not able to obtain coronary arteries from living patients, we used internal mammary arteries as a surrogate of global human arterial redox dysregulation, which has been a well-described human translational model and is associated with increased cardiovascular disease risk.^27,67,68^ Yet, this reflects only patients with severe CAD, which poses a caveat to the generalizability of our findings to broader populations. Furthermore, the surgical acquisition of samples for the OxHVF cohort means that we are not able to replicate the sizes seen in larger cohorts focused on human genetic data, which has implications for our ability to generate robust GRSs. For this reason, to improve our statistical power, dominant models of externally validated variants were used in the genetic studies in this work. This study is also limited by the small number of outcome events in the study cohort, which prevented the ability to perform subgroup analyses including between diabetics and nondiabetics. However, despite the reduced statistical power, the study was able to demonstrate an association of Lp(a) with cardiovascular mortality (independent of ApoB and other covariates including diabetes), highlighting the extent of this effect. It is also noteworthy that the Lp(a) thresholds used in this study were lower than those commonly used in primary prevention settings. Furthermore, owing to the skewed distribution of Lp(a) levels in the patient population, we were limited by the small number of patients with very-high Lp(a) levels in this study. This could have contributed to the relatively low effect sizes observed in vascular O_2_^.−^ production. Nevertheless, small changes in redox state can trigger large global changes in redox-sensitive transcription at a tissue level, which may explain the 3.3-fold increase in the risk for cardiovascular events in the high Lp(a) group, which is dependent on vascular O_2_^.−^.^67^ Although Lp(a)-induced dysregulation of redox state in the grafts used for coronary artery bypass graft could lead to higher rates of graft failure, this study was not powered to detect such differences.

In conclusion, this study is the first in humans to demonstrate that Lp(a) has a direct, and ApoB-independent, role in dysregulating redox-sensitive inflammatory signaling in the arterial wall, an effect associated with significantly elevated risk for cardiovascular mortality in patients with advanced atherosclerosis. This is a significant step in the effort to understand the pathways through which Lp(a) increases cardiovascular risk and motivates further mechanistic investigations as novel Lp(a)-lowering therapies enter clinical practice.

## Article Information

### Sources of Funding

This study was supported by the British Heart Foundation (grants FS/16/15/32047, RG/F/21/110040, and CH/F/21/90009 to C. Antoniades), the Oxford BHF Centre of Research Excellence grant RE/18/3/34214, the Oxford NIHR Biomedical Research Centre Cardiovascular Theme, an NHS (National Health Service) AI Award (grant ACRE-CT AI_AWARD02013), the Progetti di Rilevante Interesse Nazionale (grants PRIN 2017K55HLC and PRIN 20227KTSAT to G.D. Norata), Ricerca Finalizzata, the Ministry of Health (grant RF-2019-12370896 to G.D. Norata), PNRR Missione 4 (Progetto CN3-National Center for Gene Therapy and Drugs Based on RNA Technology to G.D. Norata), PNRR Missione 4 (Progetto MUSA-Multilayered Urban Sustainability Action to G.D. Norata), PNRR Missione 6 (PNRR-MAD-2022-12375913 to G.D. Norata), and the European Commission (EUROPEAID/173691/DD/ACT/XK Nanokos to G.D. Norata).

### Disclosures

C. Antoniades declared past and active consultancy agreements with Mitsubishi Tanabe, Silence Therapeutics, Novartis, Amgen, Nodthera, Caristo Diagnostics, Novo Nordisk, E Lilly, and UCB; past grants from Sanofi and Novo Nordisk; current grants from AstraZeneca, Lexicon, and Caristo Diagnostics; and honoraria from Amarin and Covance. C. Antoniades and K.M. Channon are founders, shareholders, and nonexecutive directors of Caristo Diagnostics Ltd. C. Antoniades is the past Chair and T. Guzik is the current Chair of the British Atherosclerosis Society. G.D. Norata reports research grants from Amgen, Novartis, and Pfizer and is a consultant to Merck and Viatris. The other authors report no conflicts.

### Supplemental Material

Supplemental Methods

Figures S1–S8

Tables S1–S5

Major Resources Table

References 9,24,27,30,31,32,33,35,68–79
